# mTOR Signaling Components in Tumor Mechanobiology

**DOI:** 10.3390/ijms23031825

**Published:** 2022-02-05

**Authors:** Antonios N. Gargalionis, Kostas A. Papavassiliou, Efthimia K. Basdra, Athanasios G. Papavassiliou

**Affiliations:** 1 Department of Biological Chemistry, Medical School, National and Kapodistrian University of Athens, 11527 Athens, Greece; konpapav@med.uoa.gr (K.A.P.); ebasdra@med.uoa.gr (E.K.B.); 2 Department of Biopathology, Aeginition Hospital, Medical School, National and Kapodistrian University of Athens, 11528 Athens, Greece

**Keywords:** tumor mechanobiology, mechanotransduction, mTOR, Akt, PI3K, integrin, extracellular matrix, matrix stiffness

## Abstract

Mechanistic target of rapamycin (mTOR) is a central signaling hub that integrates networks of nutrient availability, cellular metabolism, and autophagy in eukaryotic cells. mTOR kinase, along with its upstream regulators and downstream substrates, is upregulated in most human malignancies. At the same time, mechanical forces from the tumor microenvironment and mechanotransduction promote cancer cells’ proliferation, motility, and invasion. mTOR signaling pathway has been recently found on the crossroads of mechanoresponsive-induced signaling cascades to regulate cell growth, invasion, and metastasis in cancer cells. In this review, we examine the emerging association of mTOR signaling components with certain protein tools of tumor mechanobiology. Thereby, we highlight novel mechanisms of mechanotransduction, which regulate tumor progression and invasion, as well as mechanisms related to the therapeutic efficacy of antitumor drugs.

## 1. Introduction

Mechanistic target of rapamycin (mTOR) protein kinase was firstly identified as the pharmaceutical target of rapamycin, a macrolide that was isolated from the bacterium *Streptomyces hygroscopicus* and demonstrated immunosuppressive, neuroprotective, and anticancer properties [1,2,3]. During the last three decades, mTOR has been acknowledged as one of the central mediators of cellular processes, which characterize all eukaryotic cells. mTOR is a dominant regulator of cell growth, both in cell size and number, by transmitting signals inside the cell regarding the abundance of cellular nutrients and energy sources. In this way, mTOR directs biosynthesis or deconstruction of macromolecules and supports metabolic processes of cell growth and proliferation [4].

Mechanobiology is a field that investigates how mechanical forces regulate physiological processes of the human organism, and perturbations of these events have been associated with diseases. Cancer is characterized by compressive phenomena that generate aberrant mechanical forces. The way cancer cells perceive these physical alterations induces cellular responses, which facilitate tumor growth, invasion, metastasis, and chemoresistance [5,6,7]. mTOR is at the crossroad of many of these signaling networks that regulate the physical phenotype of cancer cells and transmit extracellular mechanical signals [8,9,10]. mTOR upstream regulators involve membrane receptors, integrins, and proteins of the focal adhesion complexes, which mediate sensing and transmission of mechanical cues. Furthermore, mTOR-associated cytoplasmic kinases and phosphatases, guanosine triphosphatases (GTPases), and transcription factors are also involved in molecular mechanisms generated by aberrant physical forces.

In this review, we highlight novel mechanisms that incorporate mTOR signaling components as part of the processes of mechanosensing and mechanotransduction during carcinogenesis. We examine mTOR, upstream regulators, and downstream substrates in conjunction with documented cellular tools of mechanobiology. In this context, we aim to highlight the crosstalk of signal transduction mechanisms and provide insights for the better therapeutic efficacy of anticancer strategies.

## 2. Tumor Mechanobiology

Mechanobiology is a field that integrates biology, physics, and bioengineering to explore how cells and tissue mechanics affect cell behavior, morphogenesis, and diseases. Cellular mechanobiology investigates the processes of mechanosensing and mechanotransduction. Mechanosensing is the putative ability of a cell to sense the mechanical signals, which are provided by the microenvironment. Mechanotransduction is the biochemical process through which cells transform these extracellular mechanical inputs into intracellular biochemical reactions and elicit a respective cellular response [11].

Cells are subjected to a variety of mechanical cues, which are generated from their microenvironment, and they also experience physical forces from the surrounding tissue. These environmental cues involve forces produced by neighboring cellular structures and those produced in confined interstitial spaces, such as mechanical tension, compression, and hydrostatic pressure. Cells also experience forces applied by alterations of the extracellular matrix (ECM) stiffness and shear stress from the fluid flow (e.g., blood). However, there are distinct cellular responses to these stresses according to the type of cell, the type of tissue, the cellular context, and the way each type of cell perceives the respective cue [12].

Mechanosensing and mechanotransduction require specific cellular tools for them to develop and elicit their response. These tools are protein structures called mechanosensors, and we can find them throughout the cellular structure. There are mechanosensors at the cell membrane, such as mechanosensitive transmembrane receptors, growth factor receptors, structures that protrude from the plasma membrane such as the primary cilia, and protein complexes that mediate cell-to-cell and cell-to-ECM interactions. Additional mechanosensitive tools are mechanosensitive ion channels, proteins of the ECM, the cytoskeletal components, and nuclear structures involving the chromatin itself [13]. Focal adhesions and adherens junctions include several mechanosensor proteins such as *α*-catenin, *β*-catenin, integrins, von Willebrand factor (vWf), talin, vinculin, p130 Crk-associated substrate (cas), focal adhesion kinase (FAK), and Src kinases. Piezo and transient receptor potential (TRP) channels are the main representatives of the force-induced ion channels [12,14].

### The Role of ECM

Mechanical stimuli influence cancer growth [5]. Mechanosensitive protein structures are dysregulated and contribute to tumor progression. Alterations of ECM stiffness and the tensional state of the fibronectin fibers are correlated with cell signaling events and the progression of tumorigenesis [14]. ECM properties influence all the hallmarks of cancer by promoting self-sufficient growth, tissue invasion and metastasis, sustained angiogenesis, limitless replicative potential, evasion of apoptosis, and insensitivity to growth inhibitors [14]. It seems that cancer cells exploit increased stiffness of the ECM and increased adhesion dynamics in order to generate and respond to positive growth signals, while they “tend to ignore” apoptotic signals from the soft ECM [15]. Only malignant cancer cells have the capacity to respond to matrices of varying densities. Even inside a group of cells of the same origin, there are different biomechanical properties according to the invasive potential of the cells [16]. Application of disturbed mechanical forces and stiffening of ECM cause increased intracellular tension, increased actomyosin contractility, expansion of the tumor, and modified gene expression [17]. Substrate stiffness has been linked to increased proliferation, promotion of epithelial-to-mesenchymal transition (EMT), and resistance of ovarian cancer cells to chemotherapy [18]. We now know that mechanical load alters gene expression in breast cancer cells by promoting the expression of mechanically induced genes such as Yes-associated protein (Yap) and zinc finger E-box-binding homeobox (Zeb) transcription factors. Mechanical strain also increases resistance to chemotherapeutic drugs and increases metastases to the skull and angiogenesis in MDA-MB-231 breast cancer cells [19]. Nevertheless, the response of cancer cells’ behavior to alterations of the rigidity of ECM depends on the experimental model. Increased stiffness seems to cause invasiveness of cancer cells in 3-dimensional models. On the other hand, changes in the ECM stiffness do not significantly affect the mechanical status in 2-dimensional cultures [20]. Therefore, there is a compelling need for highly sophisticated in vitro experimental systems to investigate alterations of the matrix substrates.

According to the previous context, there is a mechanical landscape of tumor growth and metastasis that has been described. At the primary tumor site, cancer cells undergo application of compressive and mechanical stresses from the hyperproliferation of cancer cells, disturbed angiogenesis, and increased interstitial forces. These events, along with ECM stiffening and altered topography, facilitate the breach of the basement membrane by stromal cells, mainly cancer-associated fibroblasts (CAFs) and macrophages. Cancer cells migrate to the blood vessels, where they are subjected to blood shear stress and increased mechanical force due to abnormalities in the tumor vasculature. In the end, circulating tumor cells are trapped within narrow blood capillaries and extravasate to form the metastatic niche in secondary locations [5,21,22].

## 3. mTOR Biology

### 3.1. mTOR Structure and Functions

**Figure 1:**
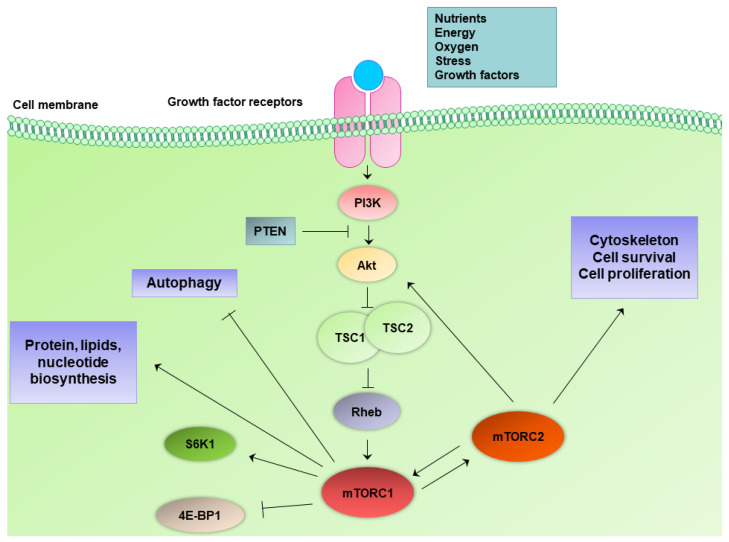
mTOR signaling pathway. mTORC1 and mTORC2 complexes with their respective upstream regulators and downstream components. Nutrient availability and growth factors induce mTOR pathway activation to suppress autophagy, mediate biosynthesis of proteins, lipids, and nucleotides, regulate cytoskeleton, cell survival, and proliferation. mTORC1, mechanistic target of rapamycin complex 1; mTORC2, mechanistic target of rapamycin complex 2; PI3K, phosphoinositide 3-kinase; PTEN, phosphatase and tensin homolog; S6K1, ribosomal protein S6 kinase beta-1; TSC, tuberous sclerosis complex; 4E-BP1, eukaryotic initiation factor 4E-binding protein 1.

Mammalian target of rapamycin, or recently known as mechanistic target of rapamycin (mTOR), is a serine/threonine protein kinase of 289 kDa molecular weight that belongs to the phosphoinositide 3-kinase (PI3K)-related protein kinases (PIKK) family. mTOR is the catalytic subunit of two distinct protein complexes, mTOR complex 1 (mTORC1) and mTOR complex 2 (mTORC2). The two complexes are associated with different protein substrates, mediate distinct signal transduction networks, and, therefore, play different roles in cellular homeostasis (Figure 1) [23]. The mTOR common domain between the two complexes contains clusters of huntingtin, elongation factor 3, a subunit of protein phosphatase 2A and TOR1 (HEAT) repeats at the N-terminal end, the FRAP, ATM, and TRRAP (FAT) domain, the FKBP12–rapamycin binding (FRB) domain, the catalytic kinase domain, and the C-terminal FATC domain.

Except for mTOR core domain structures, mTORC1 complex contains mammalian lethal with SEC13 protein 8 (mLST8 or else known as G*β*L), DEP-domain-containing-mTOR-interacting protein (DEPTOR), which is an endogenous inhibitor of mTORC1 activity, and regulatory-associated protein of mTOR (RAPTOR), which is the defining subunit of mTORC1 [23,24,25]. RAPTOR is an evolutionarily conserved protein that forms a complex with mTOR. RAPTOR can positively regulate nutrients-induced signaling and can also negatively regulate mTOR kinase activity [26]. RAPTOR can recruit proline-rich AKT substrate 40 kDa (PRAS40), which is a second mTORC1 endogenous inhibitor [27]. mTORC1 is sensitive to rapamycin and is a key mediator of the cellular metabolic responses. mTORC1 mediates the abundance of nutrients and regulates autophagy, protein, lipid, and nucleotide biosynthesis, as well as glucose metabolism. mTORC1, specifically, activates a downstream metabolic process by repressing autophagy and activating glucose metabolism, protein, lipids, and nucleotide synthesis [23]. mTORC1 exerts these actions by phosphorylating downstream protein substrates. mTORC1 directly phosphorylates p70 S6 kinase 1 (S6K1) and eukaryotic initiation factor 4E-binding proteins (4E-BPs), which activate protein synthesis. mTORC1 also regulates a series of transcription factors to mediate the biosynthesis of additional macromolecules, such as lipid-related sterol regulatory element-binding protein 1/2 (SREBP1/2) and peroxisome proliferator-activated receptor-γ (PPARγ), stress-related activating transcription factor 4 (ATF4), autophagy-related transcription factor EB (TFEB), energy-related hypoxia-inducible factor 1α (HIF1α), mitochondria-related yin–yang 1 (YY1) and PPARγ coactivator 1α (PGC1α) [28,29,30,31]. mTORC1 also has upstream regulators, which activate it when there is availability of nutrients and energy. These upstream regulators are Rag guanosine-5’-triphosphate (GTP)ases, which anchor mTORC1 to the lysosome when there is availability of amino acids, the small GTPase Rheb in its GTP-bound state, which activates mTORC1, and tuberous sclerosis complex (TSC) complex, which acts as a GTPase-activating protein (GAP) for Rheb. GTP status of Rheb is enhanced by growth factors and suppressed by low energy resources or hypoxia [32,33,34,35].

mTORC2 complex is insensitive to rapamycin, opposite to mTORC1. mTORC2 also contains mLST8, the distinct scaffolding protein RICTOR, mitogen-activated protein kinase (MAPK)-interacting protein 1 (mSIN1), DEPTOR, and protein associated with rictor 1 or 2 (PROTOR1/2) [23,36,37]. The mTOR/RICTOR complex mediates Akt phosphorylation on Ser^473^ and also enhances Akt phosphorylation on Thr^308^ by phosphoinositide-dependent kinase-1 (PDK1) [38]. mTORC2 regulates the rearrangements of the cytoskeleton and activates pathways of survival and proliferation [23]. mTORC2 has different protein substrates than mTORC1 and phosphorylates Akt, protein kinase Cα (PKCα), and serum- and glucocorticoid-induced protein kinase 1 (SGK1) [39,40]. Akt suppresses glycogen synthase kinase 3*β* (GSK3*β*) and tuberous sclerosis complex 2 (TSC2), which in turn suppresses mTORC1, thereby forming a mTORC2/TSC2/mTORC1 functional interaction. Overall, Akt forms a link between mTORC2 activation that ultimately leads to mTORC1 activation. mTORC2 also regulates forkhead box transcription factors 1/3a (FOXO1/3a) through Akt [41]. mTORC2 main upstream regulators are growth factors through the PI3K pathway [42,43].

Recently, an alternative mTOR complex has been identified. MTOR-associated protein, eak-7 homolog (mEAK-7), is a positive regulator of mTOR signaling and functions through the S6K2/4E-BP1 axis. This alternative mTOR signaling regulates cell proliferation and migration [44]. In human lung cancer cells, mEAK-7 is targeted by microRNA-1911-3p to suppress mTOR signaling [45]. Both mEAK-7 and mTOR signaling components are upregulated in non-small-cell lung carcinoma primary tumors and metastatic lymph nodes. mEAK-7 interacts with DNA-dependent protein kinase catalytic subunit isoform 1 (DNA-PKcs) to regulate S6K2 in cancer cells [46].

### 3.2. mTOR in Cancer Development and Progression

Although tumors present a shortage of nutrients due to their underdeveloped vasculature, mTOR is overactivated in most human tumors and promotes tumor growth, proliferation, and metastasis [47,48]. At first, we thought that the mTOR-encoding gene is rarely mutated. However, novel approaches highlight up to 33 mTOR-encoding gene mutations that provoke hyperactivation of the mTOR pathway. These mutations occur in different cancer types, and they mostly reduce binding to DEPTOR [49].

mTORC1 is activated in cancer by a variety of upstream regulators, which involve gene mutations and amplifications, epigenetic and posttranslational modifications [50,51]. mTORC1 is activated by activating mutations and amplifications of oncogenes, such as receptor tyrosine kinases (RTKs), PI3K/Akt pathway, Ras-, and Raf-driven MAPK pathway. mTORC1 is also activated by loss-of-function mutations of tumor suppressor genes phosphatase and tensin homolog (PTEN), TSC1/2, neurofibromatosis type 1 (NF1), liver kinase B1 (LKB1)/STK11, and adenomatous polyposis coli (APC) [48,52]. When mTORC2 is upregulated, it can also activate mTORC1 through the Akt/TSC/Rheb/mTORC1 axis [48,53]. Except for the mTORC2-specific regulation of the Akt oncogene, mTORC2 participates in cancer pathogenesis through the regulation of cytoskeletal rearrangements that drive cell invasion and motility [54,55]. mTOR is the central hub that also mediates dynamic signaling crosstalk and metabolic flux between cancer cells and cells from the tumor microenvironment. Alterations in cancer cell metabolism and changes of intracellular nutrients contribute to hyperactivation of mTORC1 and promotion of cancer cell properties [56]. Furthermore, the mTOR pathway is involved in mechanisms of therapeutic resistance. mTOR targeting is continuously investigated as part of therapeutic combination treatments and to overcome therapeutic resistance [57].

## 4. mTOR Components Involved in Mechanotransduction in Cancer Cells

**Figure 2:**
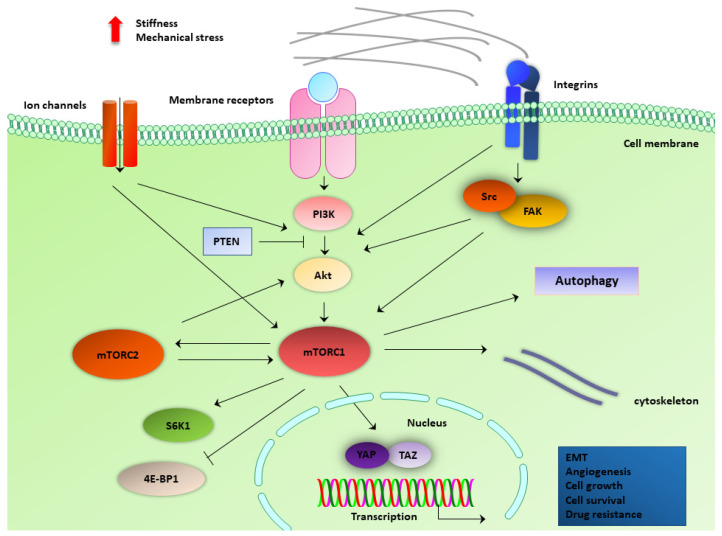
mTOR signaling components involved in mechanotransduction of cancer cells. mTOR pathway integrates signals from alterations of extracellular mechanical cues (e.g., mechanical stress, ECM stiffness). mTOR, along with its upstream regulators (PI3K, PTEN, Akt) and downstream substrates (S6K1, 4E-BP1), communicates with mechanotransduction pathways and tools, such as mechanosensitive ion channels, membrane receptors and ligands, integrins, proteins of focal adhesions (e.g., FAK and Src), components of the cytoskeleton, and mechano-induced transcription factors (YAP and TAZ). This interplay regulates autophagy and promotes EMT, angiogenesis, cell growth, cell survival, and drug resistance. EMT, epithelial-to-mesenchymal transition; FAK, focal adhesion kinase; mTORC1, mechanistic target of rapamycin complex 1; mTORC2, mechanistic target of rapamycin complex 2; PI3K, phosphoinositide 3-kinase; PTEN, phosphatase and tensin homolog; S6K1, ribosomal protein S6 kinase beta-1; 4E-BP1, eukaryotic initiation factor 4E-binding protein 1; YAP, Yes-associated protein.

During the last decade, there have been studies that investigate the behavior of mTOR signaling components under mechanical load in several systems of physiology and disease [58,59,60]. Mechanical stresses from the microenvironment regulate the process of autophagy through mTOR signaling. MTOR is in the center of intrinsic signaling pathways, which are facilitated by cytoskeletal components and mechanosensitive protein complexes [61]. Application of mechanical load activates mTOR and leads to growth of skeletal muscle cells and regulation of cartilage tissue homeostasis [58,60]. mTOR has also been implicated in the mechanobiology of aging [62]. Therefore, we discuss data that highlight the activity of mTOR and its associated signaling molecules, as well as mTOR-associated processes in conjunction with force-induced signaling mechanisms (Figure 2).

### 4.1. Force-Regulated Autophagy

Autophagy is an evolutionary conserved intracellular process that involves the degradation and recycling of cellular structures/products to regulate metabolism and homeostasis. Autophagy is triggered by a variety of stress-related stimuli, such as damage of organelles, lack of nutrients, and presence of abnormal proteins. mTOR plays a pivotal role during the process of autophagy. Activated mTORC1 leads to suppression of autophagy through phosphorylation of autophagy-related protein (ATG). In cancer, autophagy has a dual role either as a tumor-promoting or tumor-suppressing process [63].

It seems that mechanical stresses are associated with autophagy through the crosstalk between mechanotransduction and autophagy proteins (e.g., mTOR-mediated mechanical signaling) [61,64]. An additional way of autophagy/mechanotransduction interaction is the cooperation or competition between the two networks for cytoskeletal components and phospholipid membranes [61,65]. Application of compressive stress induces the formation of autophagosome through mTOR both in mammalian and single-celled Protista [66]. Mechanical stimulation upregulates autophagy and phosphorylation of most of the mTOR signaling components, such as Akt, 4E-BP1, and S6K1 [67]. Mechanical forces can regulate autophagy specifically through mTORC1 and homeostasis proteins of the cytoskeleton, such as the cochaperone BAG3 [66]. Compressive stress is also able to upregulate autophagy and promote invasion in HeLa cells by increasing the secretion of matrix metalloproteinase 2 (MMP2) and paxillin turnover [68]. In addition, another type of mechanical force, shear stress, evokes autophagy that protects HeLa cells from apoptosis [69].

### 4.2. PI3K/Akt Pathway

PI3K has been recently considered as a key signaling hub in mechanotransduction [8]. For example, PI3K/Akt pathway is activated as a response to cyclic stress in mouse osteoblast cell line MC3T3-E1 [70]. During tumorigenesis, PI3K/Akt signaling is activated by mechanosensitive integrin stimulation and mediates integrin-dependent attachment, as well as spreading cancer cells [71]. PI3K signaling is specifically activated in premalignant and malignant mammary tissues and cells. Akt is further upregulated by tension-induced growth factor signaling in mucoepidermoid carcinoma (MEC) cells following ECM increased stiffness [72]. Furthermore, PI3K is activated and mediates phosphatidylinositol (3,4,5)-triphosphate (PIP3) phosphorylation through a vinculin–talin–actin complex. This complex is assembled by ECM stiffness in mammary cells in vitro and in vivo [73]. These data suggest a role for integrin mechanotransduction in PI3K-mediated breast tumor invasion.

PIK3CB catalytic subunit induces transformation of breast cells during mammary tumorigenesis, and it also upregulates YAP and TAZ mechano-induced transcription factors [74]. In pancreatic cancer cells, cell migration is positively associated with the application of solid/compressive stress. Cell migration is facilitated through Akt/cAMP-responsive element-binding protein 1 (CREB1) pathway activation, which regulates *growth differentiation factor 15* (*GDF15)* gene expression and, particularly, upregulates *GDF15* expression as a response to compressive stress [75]. In a large-scale proteomic assay that was employed to monitor mechanotransduction pathways in pancreatic cancer, mTOR components were downregulated as a response to mechanical stress. On the other hand, PI3K/Akt signaling components are activated under mechanical stress in the same type of cells [76]. This discrepancy could be explained by the fact that while PI3K/Akt activation is induced by mechanical stress and promotes oncogenic features, mTOR repression may induce autophagy activation and ER stress response to promote cell survival and under stress [76].

### 4.3. ECM Stiffness-Induced Mechanisms

Alterations of the ECM stiffness occur during the growth of cancer cells and tumor progression. Integrins are the ECM receptors that correspond to mechanical stimuli, and downstream mTOR signaling components integrate these signals [10]. Mechanotransduction is induced by these alterations and involves the regulation of respective signaling pathways in cancer cells and cells of the tumor microenvironment, such as CAFs [77]. Compressive stress is an alternative type of force that induces glycolysis in stromal cancer-associated fibroblasts (CAFs) of breast cancer cells. This event promotes epithelial-to-mesenchymal transition (EMT) and angiogenesis, suggesting that cells forming the breast tumor microenvironment are also prominent to mechanical stimulation [78]. Mesenchymal stromal stem cells (MSCs) are recruited by cancer cells to help them promote tumor progression. Mechanical stimulation, evoked by alterations in substrate stiffness, differentially modulates miRNA expression in MSCs. This differential expression of respective miRNAs converges on mTOR, which seems to be the central hub that regulates the fate of MSCs [79].

Tensin protein mediates trafficking of *α*5*β*1 integrins to lysosomes/endosomes, and this internalization recruits and activates mTORC1, therefore suggesting a mechanism through which tension and nutrient availability regulate migration and invasion of cancer cells [80]. Under stiffer substrate, activation of the PI3K/Akt/mTOR pathway is associated with the promotion of vascular endothelial growth factor A (VEGFA)-enhanced proliferation and functions of endothelial cells, ultimately facilitating angiogenesis [81]. Increased stiffness, produced by high collagen density, is associated with higher YAP, cancer stem cell (CSC), and Akt/mTOR activity in mammary tumors in vivo. These mice also developed more and larger metastases in the lungs, whereas mTOR inhibition via rapamycin diminished the tumors [82]. Corroborating findings exhibit that matrix stiffness affects the properties of CSCs in hepatocellular carcinomas. Under increased substrate stiffness, hepatocellular carcinoma cells (HCC) demonstrate enhanced features of stemness and upregulation of Akt, mTOR, 4E-BP, and SRY (sex-determining region Y)-box 2 (SOX2) phosphorylation. mTOR inhibition suppresses the stemness of HCC cells, thereby suggesting that the *β*1 integrin/Akt/mTOR/SOX2 pathway mediates stiffness-induced cancer cell stemness in HCC [83]. Likewise, miR-17-5p activates *β*1 integrin/PTEN/PI3K/Akt/matrix metalloproteinases (MMPs) pathway under increased stiffness in HCC cells and promotes matrix stiffness-induced resistance to metformin [84]. Invasion of HCC cells is also promoted by interleukin-8 (IL-8) through PI3K/Akt/integrin *β*3 axis [85]. Furthermore, mTOR not only mediates mechanotransduction from alterations of ECM stiffness but controls the degradation of ECM proteins as an alternative metabolic pathway. When tumor epithelial cells are deprived of amino acids, mTOR repression rewires metabolism to use amino acids to form the degradation of ECM proteins in favor of the starved cells [86].

### 4.4. Mechanosensitive Membrane Protein Complexes and Ligands

Proteins of the focal adhesions are tools of mechanotransduction. Focal adhesion kinase (FAK) is a core mechanotransduction protein. FAK acts upstream of mTORC1 signaling. However, mTORC1 has also been identified as an upstream regulator of pressure-stimulated FAK phosphorylation, therefore forming a reciprocal feedback loop. Triggering of mechanotransduction also changes the subcellular localization of mTORC1, whereas mTORC1 and FAK coordinated activity enhances the proliferation of MSCs [87]. Integrin signaling mediates a downstream pathway that consists of FAK, Src, PI3K, Akt, and mTOR to regulate rRNA transcription and, therefore, cell growth [88]. Caveolin-1 (Cav-1) is a main protein of caveolar membrane domains. Cav-1 activates PI3K/Akt/mTOR pathway under low shear stress and promotes motility and a metastatic phenotype of MDA-MB-231 breast cancer cells, therefore plays a role in disseminated cells to blood and lymphatic vessels [89]. Periostin is a ligand for integrin signaling in epithelial cells. Periostin biology has been implicated in cancer development and progression. Periostin acts as a paracrine ligand that activates the mTOR pathway, cellular proliferation, and migration [90]. In renal cell carcinoma, periostin promotes EMT through IKL/Akt/mTOR signaling pathway [91]. PC1 and PC2 are mechanosensitive proteins that form complexes at the plasma membrane and the primary cilia. PC1 and PC2 regulate the mTOR pathway components, such as S6K1, and are associated with aggressive phenotypes in CRC [92].

### 4.5. Mechanosensitive Ion Channels and Cytoskeletal Components

Ion channels that respond to mechanical stimuli are critical tools of mechanotransduction in eukaryotic cells. Piezo1 is a typical representative of ion channels that respond to membrane deformations that produce physical cues. Piezo1 and other selective mechano-induced ion channels respond by allowing calcium influx, which is a notable signaling hub for transmitting mechanical forces. Piezo1 is involved in cancer progression, especially during invasion and metastasis [93]. Piezo1 expression is elevated in human prostate cancer tissues and cell lines. Akt and mTOR are activated and mediate Piezo1 activity, which promotes cell cycle progression in prostate cancer cells [64]. Transient receptor potential (TRP)V4 is also a family of stretch-activated ion channels. TRPV4 are activated under low mechanical confinement along with Akt/mTOR activation and drive cytoplasmic localization of p27^Kip1^, thus facilitating entry to the S phase and proliferation in cancer cells [94]. PC2 is a mechanosensitive cation-selective ion channel that belongs to the TRPP family of proteins. PC2 overexpression results in upregulation of the mTOR pathway in SW480 colorectal cancer (CRC) cells. PC2 aberrant expression is also associated with poor overall survival and aggressive tumor parameters in CRC human tissues [92]. In addition, low shear stress increases the density of the V–H^+^-ATPase proton pump, *β*1 integrin, endosome, lysosome and also induces mTORC2 activation [95].

Modulation of mTORC2 activity causes alterations of actin distribution and changes in focal adhesion. Furthermore, the association of filamin A with RICTOR has been highlighted by mass spectrometry. RICTOR colocalizes with and phosphorylates filamin A in GBM cells, thereby suggesting a direct link between mTOR signaling components with cytoskeletal elements in GBM [96]. mTORC2, along with phospholipase D2 (PLD2), mediate a mechanosensory feedback mechanism under mechanical tension through which neutrophils organize cell polarity and motility [97].

### 4.6. Crosstalk between the MTOR and Hippo Pathway

**Figure 3:**
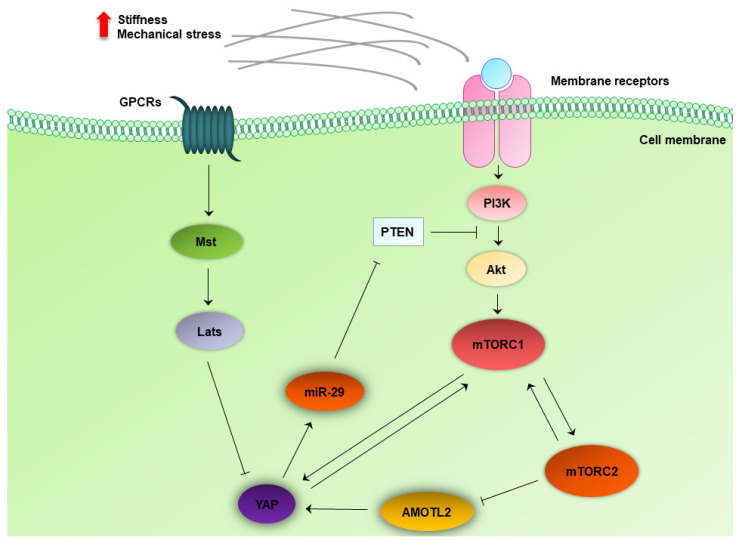
Crosstalk between the mTOR and Hippo pathway in cancer cells. Yap acts as the effector of the Hippo pathway and regulates PI3K/Akt pathway through miR-29 and PTEN. There is mutual crosstalk between YAP and mTORC1. Additionally, mTORC2 negatively regulates AMOTL2, which, in turn, activates YAP. AMOTL2, angiomotin-like protein 2; GPCRs, G-protein-coupled receptors; miR-29, microRNA-29; mTORC1, mechanistic target of rapamycin complex 1; mTORC2, mechanistic target of rapamycin complex 2; Mst, macrophage-stimulating; Lats, large tumor suppressor kinase; PI3K, phosphoinositide 3-kinase; PTEN, phosphatase and tensin homolog; YAP, Yes-associated protein.

The Hippo-YAP signaling integrates processes of cellular and organismal metabolism. mTOR responds to the presence of amino acids and to insulin/insulin growth factor 2 (IGF2) signaling to suppress autophagy and YAP [98]. YAP facilitates crosstalk between the Hippo and the PI3K/Akt pathway by suppressing PTEN (Figure 3) [99]. This crosstalk regulates cell growth and organ size [100]. It is already known that the two mechanoresponsive transcription factors, YAP and TAZ, are regulated by the Hippo pathway, and when activated, they exert oncogenic actions in solid tumors and glioblastogenesis [101]. A novel crosstalk between the Hippo pathway and the mTOR pathway has been recently identified. In a genetically engineered mouse model with conditional RICTOR overexpression, angiomotin-like protein 2 (AMOTL2) was identified as the intermediate link between mTORC2 and the Hippo pathway. mTORC2 negatively phosphorylates AMOTL2 at Ser^760^. Phosphorylation/inactivation of AMOTL2 enhances transcription of YAP-targeting genes and promotes glioblastoma (GBM) growth, motility, and invasion in vitro, as well as GBM growth in vivo (Figure 3) [102]. Data from clinical specimens demonstrate that high expression of mTORSer^2448^ phosphorylation and YAP are correlated with poor overall survival in patients with glioma and show a beneficial effect of dual targeting in vitro. Since mTORSer^2448^ phosphorylation is typical for mTORC1 activation, there is a functional association of mTORC1 and YAP in gliomas [103]. Furthermore, YAP and TAZ are themselves critical regulators of mTORC1. YAP and TAZ activate mTORC1 in the presence of amino acids; therefore, they can regulate the central mTORC1 pathway [104]. Conversely, mTOR upregulates YAP in mouse and human perivascular epithelioid cell tumors, which can be present in patients with TSC. YAP enhancement and accumulation are also associated with defective degradation of the protein by impaired autophagy process in cells lacking TSC1/TSC2. YAP is both regulated by autophagy and mTOR, thus linking mechanical signaling with nutrient-associated processes [105].

Hepatoblastoma (HB)is a common liver tumor in children. During HB development, a positive feedback loop has been highlighted between YAP and mTORC1, thereby forming a vicious circle of regulation. Specific mTORC1 ablation delays YAP/*β*-catenin-induced HB development in mice and, inversely, YAP and/or TAZ silencing decreases mTORC1 activation via reduction in the amino acid transporter sodium-coupled neutral amino acid transporter 1 (SLC38A1) [106]. Experiments in 3-dimensional (3D) collagen scaffolds demonstrate that the YAP/mTOR axis is an ultimate effector of 3D culture-driven mechanotransduction and mediates cell plasticity in liver cancer cell lines [107]. YAP is also overexpressed in human lung adenocarcinoma tissues, regulates autophagy and proliferation in A549 and H1299 cancer cells, and induces activation of Akt/mTOR signaling through suppression of PTEN via the Hippo pathway [108].

## 5. mTOR Targeting and Mechanotransduction Interplay in Anticancer Treatments

mTOR has been a promising target for cancer therapy. There are compounds that have already been approved for use in the clinic, and there are even more drugs that are being tested in preclinical models and clinical trials. mTOR-targeting drugs involve analogs of rapamycin, ATP-competitive mTOR inhibitors, and dual PI3K/mTOR inhibitors [2].

As it was recently highlighted in experiments of compressive stress application in pancreatic cancer cells spheroids, increased compressive stress results in reduced drug efficacy. The proposed mechanism suggests that compressive stress reduces cell proliferation; thereby, chemotherapeutic drugs show minimum efficacy against proliferating cells [109]. ECM-attached ovarian cells in 3D cultures are resistant to PI3K/mTOR inhibition. This resistance is mediated by cell survival programs [110]. Whole exome sequencing has revealed different gene signatures between susceptible and insusceptible patients with head and neck squamous cell carcinoma (HNSCC) cell lines to *β*1 integrin (AIIB2) and epidermal growth factor receptor (EGFR) (Cetuximab) targeting. Dual inhibition of mTOR and Kelch-like ECH-associated protein 1 (KEAP1) could reduce resistance to both selective compounds [111]. mTOR signaling is upregulated in osteosarcoma [112]. Three-dimensional osteosarcoma in vitro model shows increased resistance to chemotherapy and insulin growth factor-1 receptor (IGF-1R)/mTOR targeting [113]. mTOR inhibition and knockdown of mTOR downstream effectors 4E-BP1 or S6K1 can alleviate the effect of mechanical load on MG-63 3D cultures, which is translated into mechanosensitive fibronectin III A1 (FNIII A1) variant of tenascin-C, TN-C FNIII A1 upregulation. These data imply the potential efficacy of mTOR inhibition against tumorigenic effects of mechanotransduction in osteosarcoma [114].

Transgenic mice with mammary tumors and a genetic profile of *human epidermal receptor (HER)* positive plus *PIK3CA* mutations are resistant to HER2 antibodies trastuzumab and pertuzumab. It seems that ECM and cell adhesion genes, integrin *β*1 and Src, are overactivated in these tumors. Furthermore, HER2^+^ breast cancer patients with elevated collagen II expression are poor responders to neoadjuvant anti-HER2. In vitro experiments also demonstrate that drug resistance is ECM-induced and can be reversed by inhibition of integrin *β*1/Src [115]. The *β*1 integrin/Src/Akt pathway also facilitates acquired resistance to EGFR-tyrosine kinase inhibitor (TKI) erlotinib in lung cancer cells harboring *EGFR* mutation [116]. An alternative mechanism of collagen-mediated anti-EGFR resistance in lung adenocarcinoma patients involves S6K1 upregulation. When cancer cells were treated with mTOR inhibitor, resistance to EGFR-TKI was alleviated [117]. Likewise, in brain astrocytomas, *β*1 integrin/EGFR/Akt interplay is associated with radioresistance in vitro [118]. In hepatocellular carcinoma, galectin-1 induces resistance to kinase inhibitor sorafenib through PI3K/Akt [119]. ECM-mediated integrins *α*3*β*1 and *α*11*β*1/Src/YAP1 axis also evoke resistance to PI3K/Akt and MAPK targeting in melanoma cells [120].

## 6. Conclusions

Tumor mechanobiology is now a distinct field that investigates the impact of aberrant mechanical forces on tumor development, progression, and therapy. Cancer cells communicate with their microenvironment and translate physical alterations, changes of ECM rigidity, concentration of ligands, and reprogramming of cellular architecture into corresponding cellular response through mechanotransduction [5,6,7,21]. In this context, it seems that mTOR upstream and downstream signaling components are at the crossroad of mechanotransduction processes. Given the above data, the mTOR signaling network responds to alterations of ECM composition, calcium influx through mechanosensitive ion channels, membrane receptors, and proteins of the focal adhesion complexes. PI3K/Akt/mTOR pathway also regulates mechano-induced transcription factors, which elicit the cellular response to mechanical stimulation.

Notably, several studies highlight the contribution of the mTOR pathway in mechanisms of drug resistance [2,52,105,109,110,111,112,116,117,118,120]. In these mechanisms, mTOR holds a dual role. mTOR pathway either participates in force-induced molecular mechanisms that confer resistance to applied treatment or mTOR targeting holds promise for cancer cells’ resensitization to chemotherapy, targeted therapy, or radiotherapy. Thereby, future studies should further investigate the interplay between mTOR signaling and mechanotransduction but also focus on elucidating the role of this central hub in mechanisms of drug resistance and the results of mTOR combination treatments [121,122].
